# Open Dislocation of the Scaphoid With an Associated Hamate Fracture and Fourth Metacarpal Fracture

**DOI:** 10.1016/j.jhsg.2023.10.004

**Published:** 2023-11-18

**Authors:** Cay Mierisch, Robert Wood, Jacob Pearson, Gary Ulrich, Madeleine Vergun

**Affiliations:** ∗ Department of Orthopaedic Surgery, Samaritan Health Services Good Samaritan Regional Medical Center, Corvallis, OR; † Samaritan Health Services Orthopaedic Surgery Residency, Corvallis, OR; ‡ Department of Orthopaedic Surgery and Sports Medicine, University of Kentucky College of Medicine, Lexington, KY

**Keywords:** Fourth metacarpal fracture, Hamate fracture, Open, Scaphoid dislocation, Volar

## Abstract

Scaphoid dislocation represents a rare injury with only a few case reports and limited case series reported in the literature. The majority of scaphoid dislocations result from a high-energy trauma causing hyperextension and ulnar deviation of the wrist. The severity of a scaphoid dislocation depends on the degree of periscaphoid ligamentous injury as well as the presence of concomitant injuries, such as axial carpal dissociation. The most common complication after a scaphoid dislocation is scapholunate dissociation, which emphasizes the importance of scapholunate ligament repair/reconstruction in these cases. We report a case of an open scaphoid dislocation with the associated injuries of a hamate fracture and fourth metacarpal fracture treated with an open reduction of the scaphoid, open ligamentous repair and augmentation of the involved carpal ligaments, and open reduction internal fixation of both the hamate and the fourth metacarpal fractures.

Scaphoid dislocations represent a rare injury with only a few case reports and limited case series reported in the literature since its initial description in 1903.^1^ Scaphoid dislocations result from a high-energy trauma with the wrist in hyperextension and ulnar deviation. Because of the unconventional nature of this injury, the diagnosis of a scaphoid dislocation is often initially missed, with a delay to diagnosis representing the greatest risk factor for poor outcome.^2^, ^3^, ^4^, ^5^

The severity of the scaphoid dislocation depends on the periscaphoid ligamentous injury because the scaphoid can either be partially dislocated and tethered by soft tissue or totally dislocated with no soft tissue attachments.^6^ Partial dislocations are more common, where the distal pole of the scaphoid remains attached distally by the scaphotrapeziotrapezoid ligaments and scaphotrapezial articulation.^2^ When identified early, treatment options for scaphoid dislocation have been successful and include either closed or open reduction followed by cast immobilization, K-wire fixation, and/or ligamentous repair/reconstruction.^7^^,^^8^

Although scaphoid dislocations represent extremely rare injuries within themselves, a scaphoid dislocation is even more sparse in the setting of an open injury. We report a case of an open scaphoid dislocation with the associated injuries of a hamate fracture and fourth metacarpal fracture treated with an open reduction of the scaphoid, open ligamentous repair and augmentation of the involved carpal ligaments, and open reduction internal fixation of both the hamate and the fourth metacarpal fractures. Informed consent was acquired from the patient for the publication of this case report and accompanying images.

## Case Report

**Figure 1:**
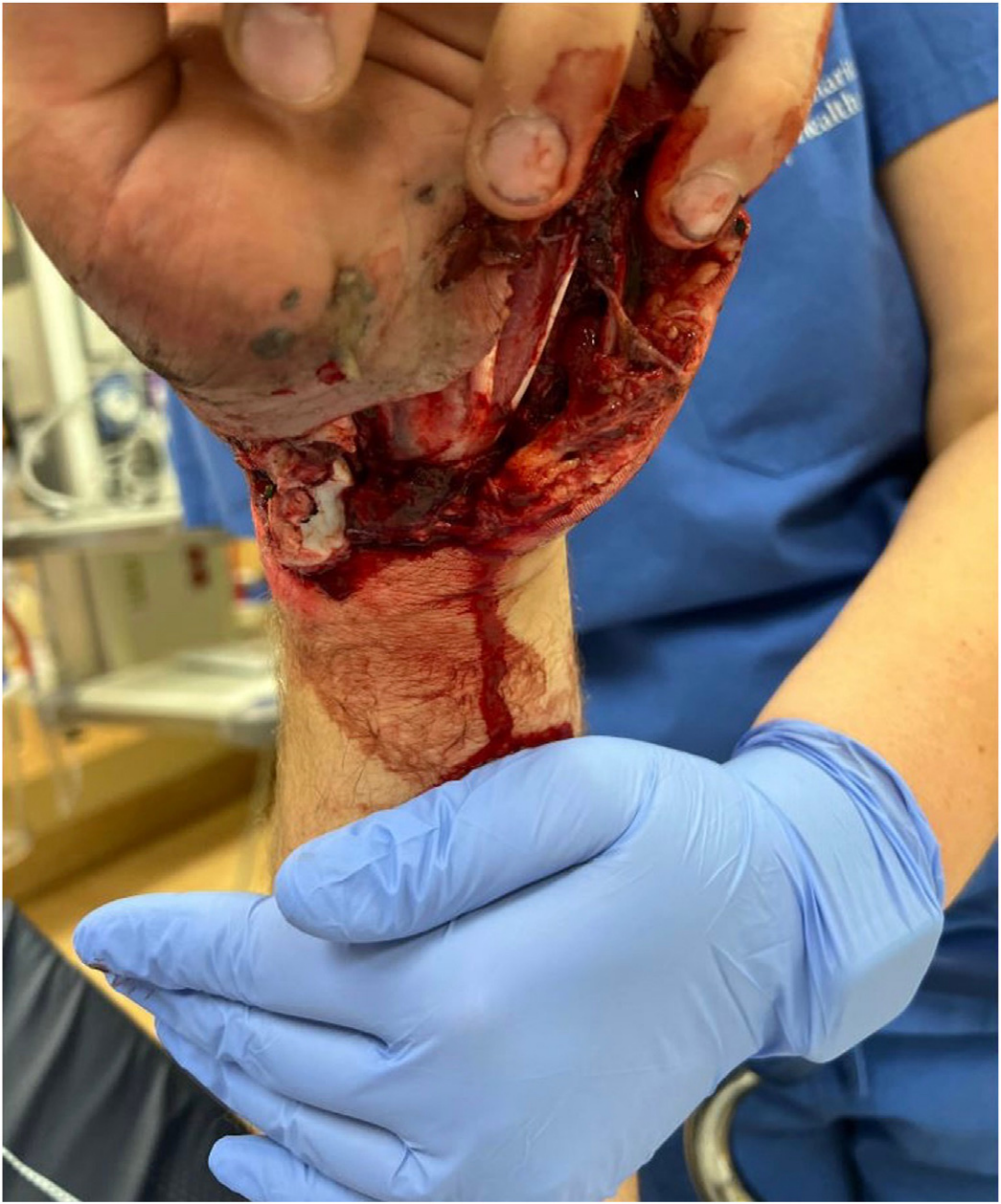
An approximately 20-cm laceration from the volar radial aspect of the proximal wrist extending distally to the volar ulnar aspect of the base of the little finger. The dislocated scaphoid is directly visualized within the laceration to be distally tethered by soft tissue attachments.

A right-handed 18-year-old man presented to the emergency department after sustaining a traumatic open left-hand injury from an all-terrain vehicle accident. The injury occurred when the patient struck his outstretched left hand on the ground in a hyperextended and ulnar-deviated position in an attempt to prevent the all-terrain vehicle from tipping over onto its left side. The patient sustained an approximately 20-cm laceration from the volar radial aspect of the proximal wrist extending distally across the carpal tunnel to the volar ulnar aspect of the base of the little finger. The scaphoid was directly visualized through the laceration to be volarly dislocated with some soft tissue attachment (Fig. 1).

**Figure 2:**
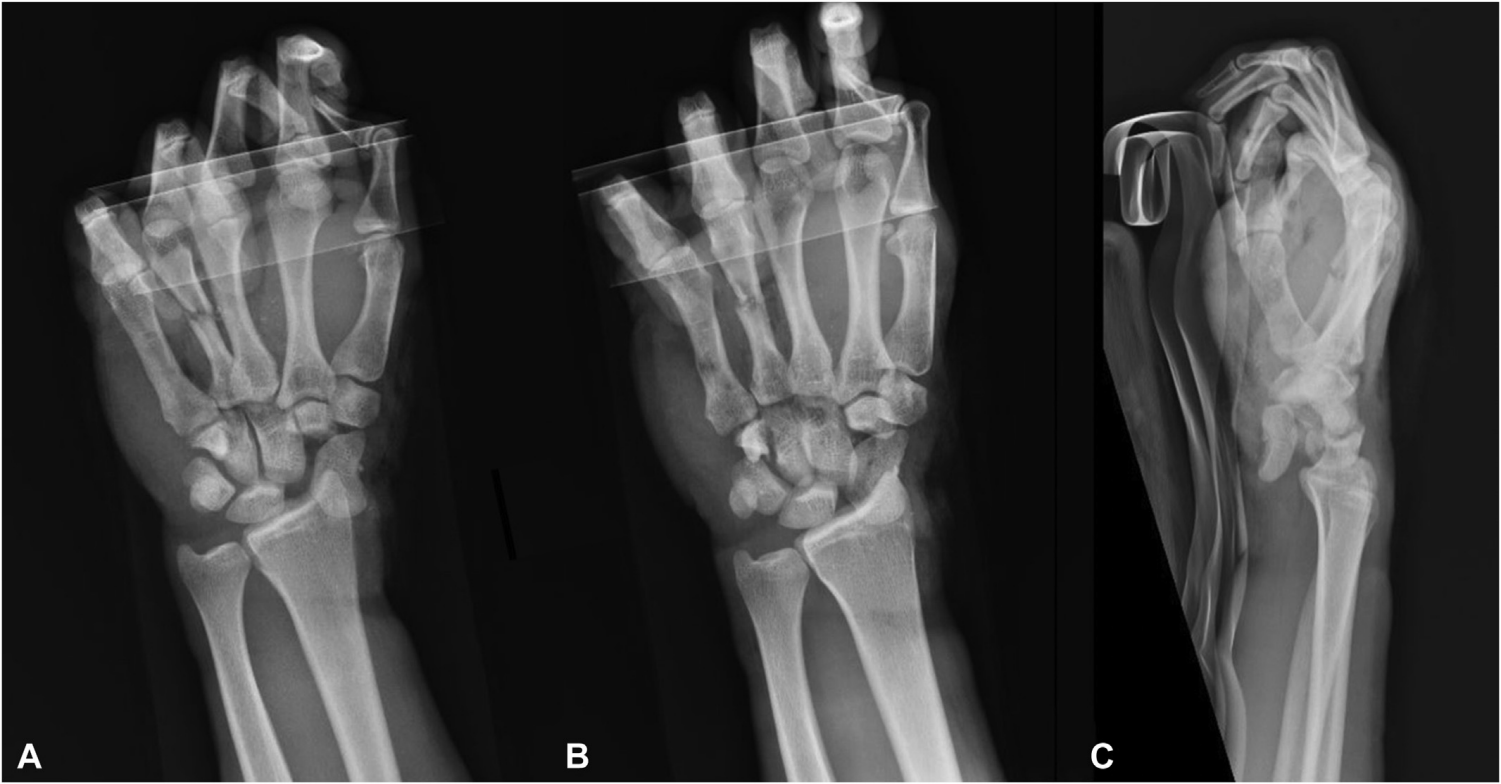
Three radiographic views of the left wrist. **A** Posterior-anterior view. **B** Oblique view. **C** Lateral view. These images demonstrate a palmar–radial scaphoid dislocation, a hamate body fracture at the base of the hook of hamate, and a fourth metacarpal midshaft fracture.

The patient was neurovascularly intact and was able to actively flex the metacarpophalangeal joints and interphalangeal joints. A visible deformity over the ulnar aspect of the dorsum of the hand was noted at the level of the fourth midshaft metacarpal. The forearm compartments were soft and compressible, and a secondary orthopedic examination was negative. Radiographs confirmed a volarly dislocated scaphoid as well as a minimally displaced midshaft fourth metacarpal fracture and a hamate body fracture at the base of the hook of hamate (Fig. 2A–C). The patient was administered 2 g of cefazolin, a Tdap injection, and pain medication and was placed in a static volar splint. Based on patient presentation, the decision was made to proceed with emergent surgical intervention.

**Figure 3:**
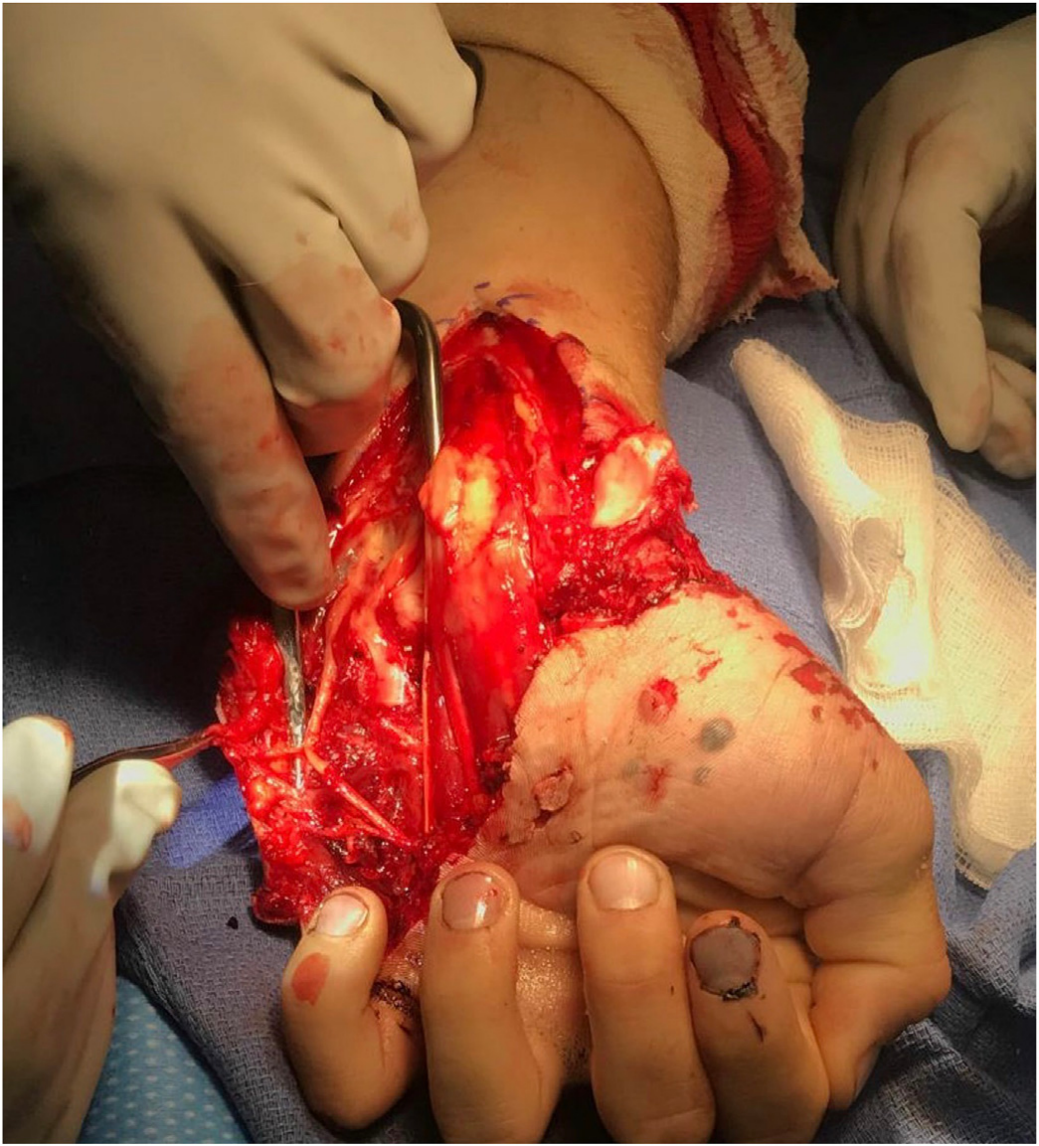
Intraoperative image depicting the surgical release of the ulnar nerve and its branches. The scaphoid is also seen dislocated.

A volar incision centered over the palmaris longus tendon was made, extending the laceration proximally. A formal carpal tunnel release was performed after visualizing a partially torn transverse carpal ligament off its radial attachment to the trapezium. Neurolysis of the median nerve and its common digital nerve branches to the first, second, and third web spaces was then performed. The median nerve and its branches were intact, but there was significant bruising of the nerves. Given the hamate fracture, Guyon canal was released as well as the motor branch of the ulnar nerve and common digital nerves to the fourth web space and little finger (Fig. 3).

**Figure 4:**
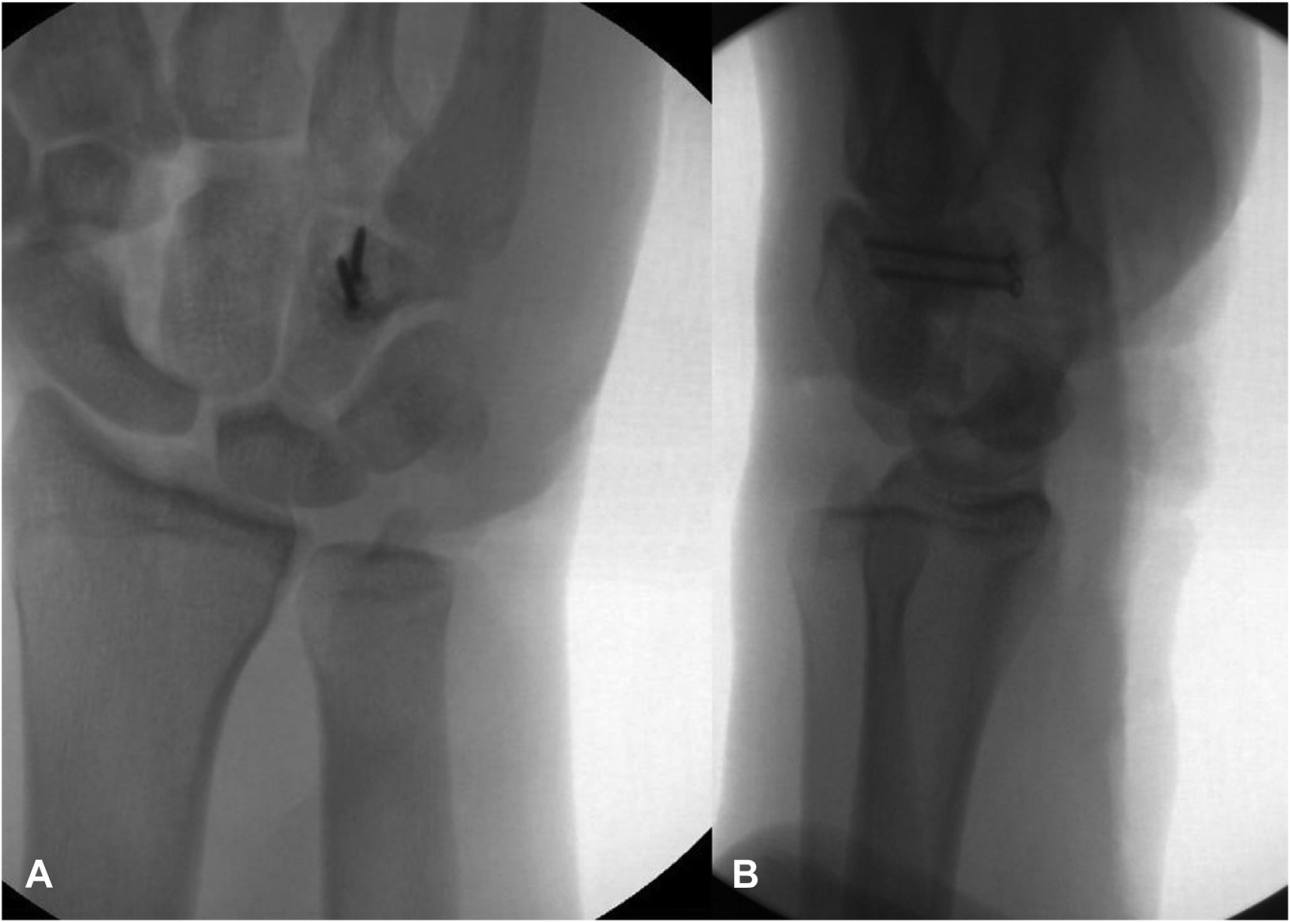
Two fluoroscopic views of the left wrist. **A** Posterior-anterior view. **B** Lateral view. These images demonstrate reduction and fixation of the hamate fracture with two interfragmentary screws as well as a reduced scaphoid into the scaphoid fossa.

Through the volar laceration, the hamate fracture was anatomically reduced and fixated with two 1.6-mm interfragmentary screws. Next, the scaphoid was reduced into its anatomic position (Fig. 4A, B). The scaphoid was found to be tethered by the volar ligaments, but the dorsal ligaments had been avulsed. The decision was made to make a dorsal midline incision to repair the dorsal ligaments and perform a posterior interosseous nerve neurectomy. During the approach, it was observed that the dorsal intercarpal ligament and the dorsal scapholunate ligament were both avulsed from their scaphoid insertions. Therefore, the decision was made to repair and augment these ligaments with swivel lock anchors, 3-0 FiberWire, and FiberTape.

The scapholunate interval was anatomically reduced with two temporary K-wires. Next, a swivel lock anchor with attached 3-0 FiberWire was placed into the proximal pole of the scaphoid and used to repair the interosseous ligament. As an internal brace for this repair, FiberTape also attached to this swivel lock was brought over the scapholunate interval and anchored to a swivel lock in the lunate. The FiberTape was then brought back over radially to be anchored to a swivel lock anchor placed in the distal pole of the scaphoid. Then, 3-0 FiberWire was attached to the anchors in the lunate and the distal pole of the scaphoid and was used to suture the dorsal intercarpal ligament back down to bone at each of these respective locations. An additional Nano Corkscrew FT anchor was placed into the triquetrum for reinsertion of the dorsal intercarpal ligament and the dorsal radiocarpal ligament. Sequential sutures were passed through the dorsal capsule to bring down the capsule and reattach the dorsal intercarpal ligament to the carpus. Carpal alignment was then examined and determined to be stable with no persistent dorsal intercalated segment instability deformity.

**Figure 5:**
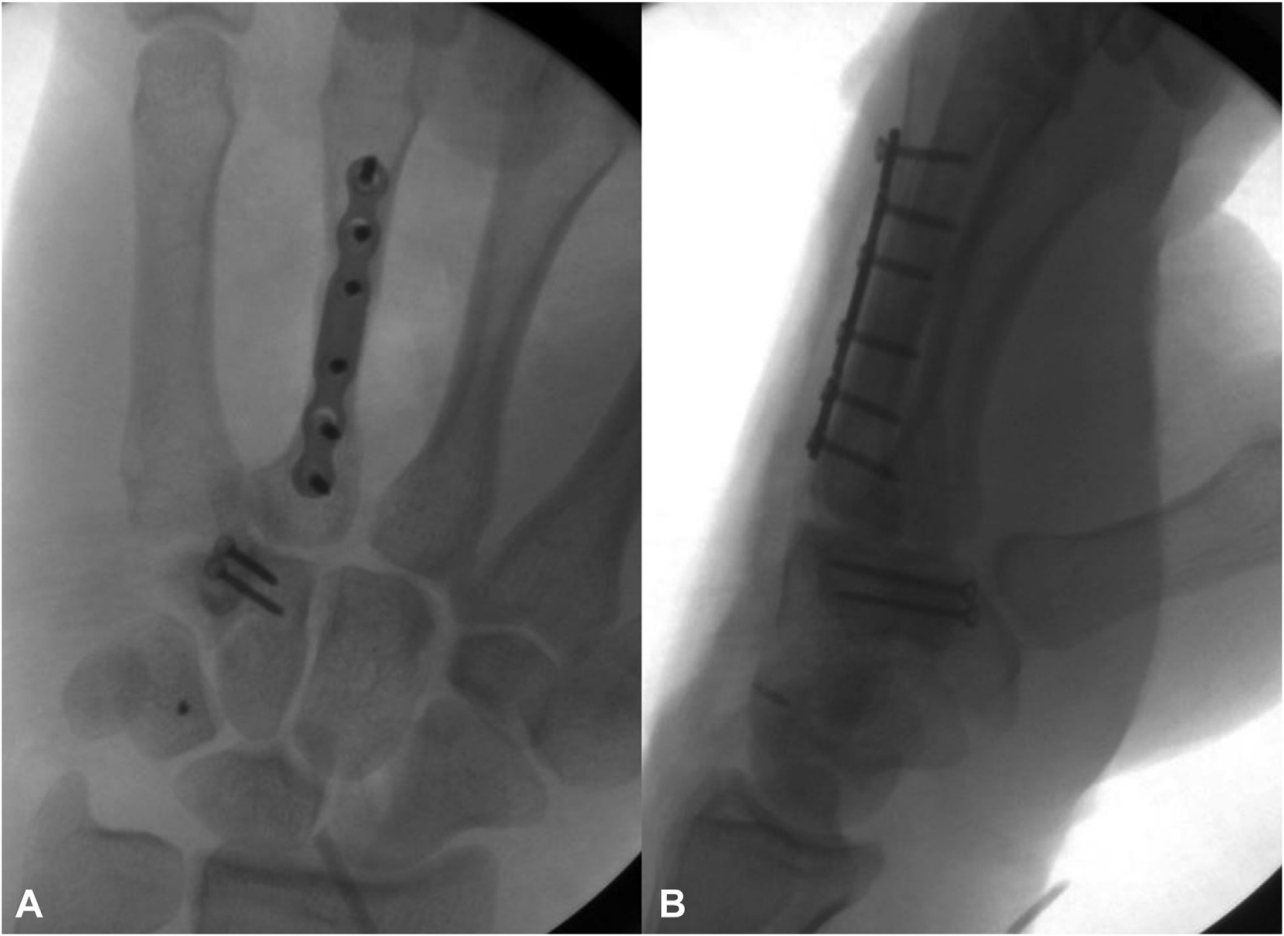
Two fluoroscopic views of the left hand. **A** Posterior-anterior view. **B** Lateral view. These images demonstrate reduction and fixation of the metacarpal fracture with a 2-mm plate. The Nano Corkscrew FT can also be visualized in the triquetrum. The scapholunate interval is optimally reduced.

**Figure 6:**
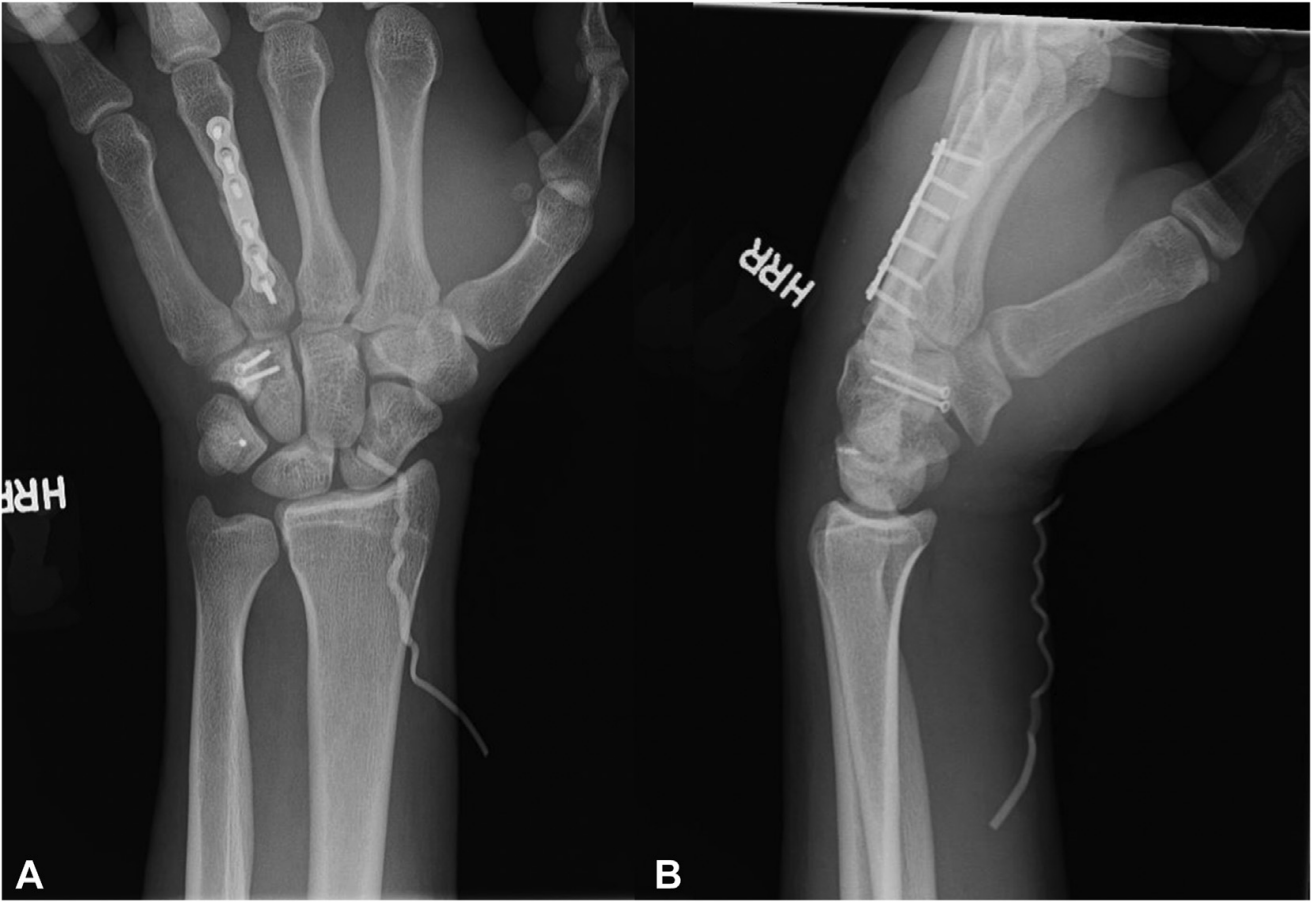
Two radiographic views of the left wrist. **A** Posterior-anterior view. **B** Lateral view. These images demonstrate optimal reduction and fixation of the patient’s multiple injuries. A vessel loop used to aid in superficial suture removal can be visualized.

Finally, to address the fourth metacarpal fracture, the dorsal skin incision was extended over the dorsal fourth metacarpal. Because of comminution at the fracture site, a near-anatomic reduction was obtained, and internal fixation was achieved with a 2-mm plate (Fig. 5A, B). The patient was placed in a well-padded volar resting splint following surgery, and the patient’s 2-week postoperative radiographs demonstrated optimal reduction and fixation (Fig. 6A, B).

At his final follow-up at 3 months after surgery, the patient continued to have limited flexion and extension of the index, middle, ring, and little finger proximal interphalangeal and distal interphalangeal joints. Moreover, the patient remained unable to flex or extend the thumb interphalangeal joint and had limited flexion and extension of the wrist. There was also diminished sensation in the radial, ulnar, and median nerve distributions of the hand. Despite these limitations, sensation and overall range of motion were improved from his initial follow-ups, and the patient was encouraged to continue with formal hand therapy. Radiographs demonstrated stable open reduction internal fixation with proper alignment of both the hamate and fourth metacarpal without signs of hardware failure. Moreover, there was no radiographic evidence of avascular necrosis of the scaphoid.

## Discussion

Scaphoid dislocation embodies a rare injury pattern, especially in the setting of an open injury. To our knowledge, only a single open scaphoid dislocation case has previously been reported in the English literature, which was caused by a wooden splinter penetrating the hand and volarly dislocating the scaphoid. In this case, the patient developed transient avascular necrosis in the scaphoid 3 months after surgery, as evidenced by increased radiodensity. However, the avascular necrosis began to resolve by 7 months after surgery. At the 9-year follow-up, the patient reported working as a full-time manual laborer with mild pain only evoked by strenuous wrist exercise.^9^

Surprisingly, avascular necrosis is not a common complication of scaphoid dislocation, even in cases of a total scaphoid dislocation.^2^^,^^6^ In our case, the scaphoid dislocation was classified as a partial dislocation and demonstrated no evidence of avascular necrosis. One potential explanation as to why avascular necrosis occurs so infrequently is that there exists undisturbed intraosseous vascular channels within the intact scaphoid bone, which enable rapid revascularization from surrounding soft tissues.^6^ In partial scaphoid dislocations, the distal soft tissue attachments likely provide adequate blood supply to prevent avascular necrosis.^10^

Leung et al^6^ further characterized scaphoid dislocations as primary versus secondary, simple versus complex, and direction based. Primary dislocations occur directly from the injury, whereas secondary dislocations result after a closed reduction of a dislocated proximal carpus. In simple dislocations, the scaphoid is only dislocated from the scapholunate and radioscaphoid articulations. In complex dislocations, further disruptions in the distal carpal row occur, including capitate, hamate, and middle–ring metacarpal base articulations. Finally, the dislocation direction with respect to the proximal pole can be pure radial, palmar–radial, palmar–straight, palmar–ulnar, or dorsal.

**Figure 7:**
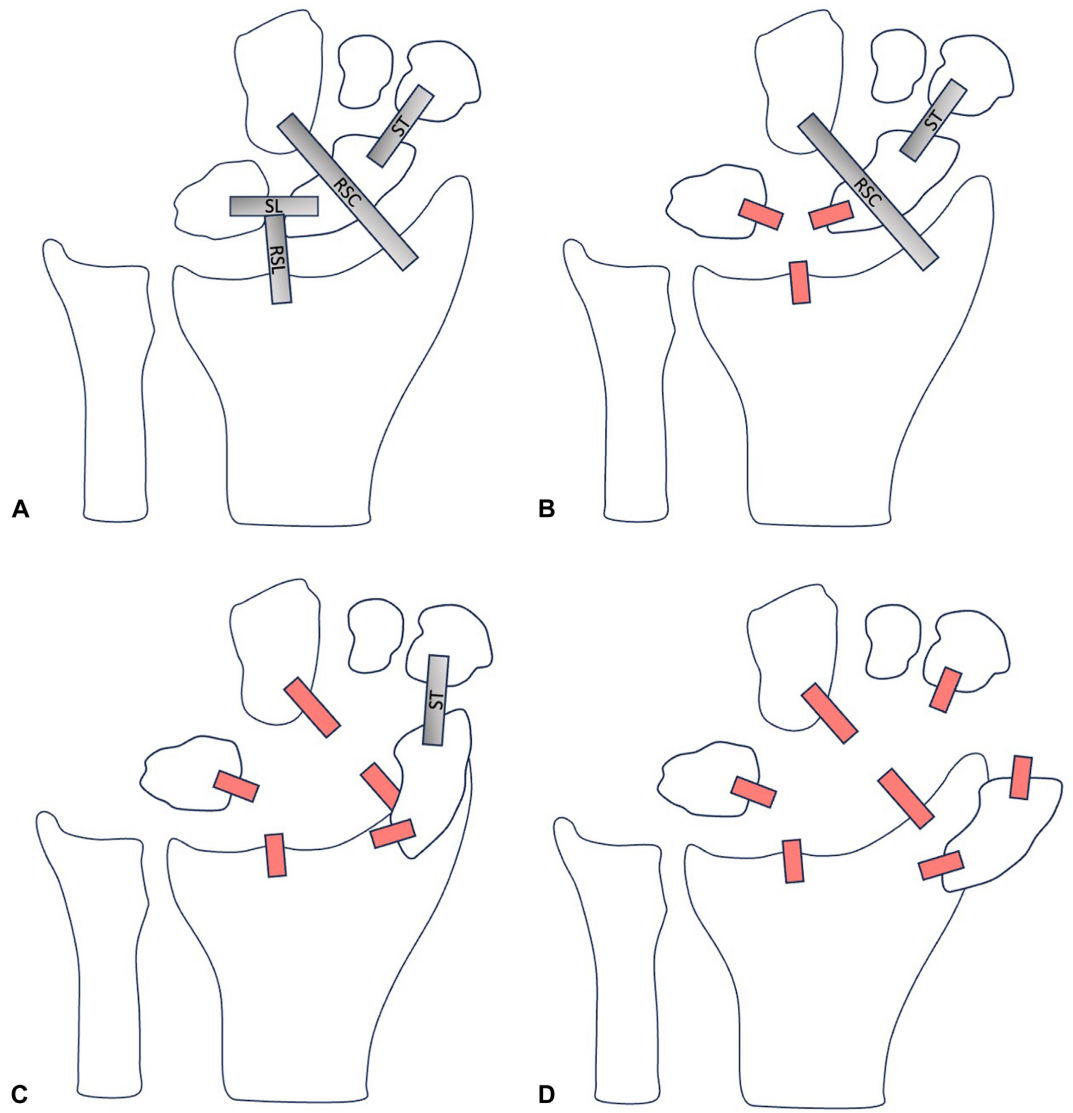
The sequence of ligamentous failure in chronological order. **A** A normal, intact wrist. **B** A torn scapholunate ligament (SL) and torn radioscapholunate ligament (RSL) resulting in scapholunate dissociation. **C** An additionally torn radioscaphocapitate ligament (RSC) resulting in a partial scaphoid dislocation. **D** An additionally torn scaphotrapezial ligament (ST) resulting in a total scaphoid dislocation.

Another method of categorizing a scaphoid dislocation is to assess it as a specific injury pattern on a spectrum of scapholunate dissociation. In other words, there are different severities of scapholunate dissociation, with a total scaphoid dislocation representing the most severe form. The sequence of ligamentous failure begins with the failure of the scapholunate ligament and the radioscapholunate ligament to cause scapholunate dissociation. Next, the radioscaphocapitate ligament fails, which results in a partial scaphoid dislocation. Finally, the scaphotrapezial ligament fails to cause a total scaphoid dislocation (Fig. 7A–D).^6^^,^^11^

**Figure 8:**
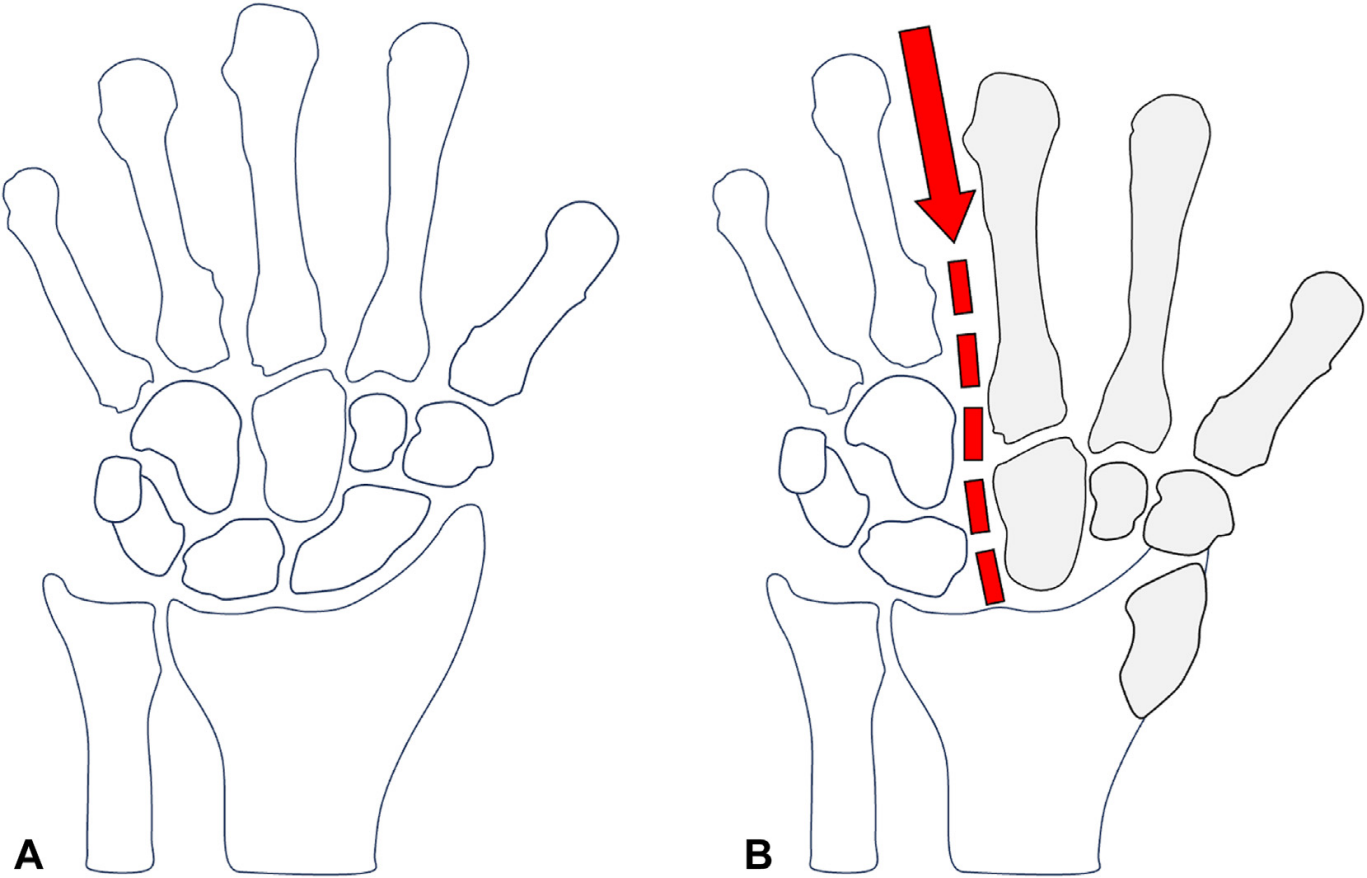
A comparison of two wrists. **A** A normal wrist. **B** A wrist with axial carpal dissociation indicated by the disruption of the capitohamate and middle–ring metacarpal base joints because of proximal migration of the radial carpus, inducing a scaphoid dislocation. The red arrow and dotted line represent the axial force transmission causing this injury pattern.

Another scaphoid dislocation injury pattern that can occur is a scaphoid dislocation with concomitant axial carpal dissociation.^12^^,^^13^ In axial carpal dissociation, the capitohamate and the middle–ring metacarpal base joints are disrupted, resulting in proximal migration of the capitate and radial metacarpals (Fig. 8A, B). Both Richards et al^14^ and Connell and Dyson^15^ proposed that this injury pattern occurs because of an axial force transmitted through the metacarpals, which then causes the capitate to abut against the scaphoid and cause its dislocation when the hand is in hyperextension and ulnar deviation. When assessing the injury pattern in our patient, there was a fourth metacarpal fracture, a hamate fracture, and a scaphoid dislocation. It is intriguing to speculate whether our patient’s injury pattern resulted from an axial force dissipation comparable to one that would have caused axial carpal dissociation. However, in our case, the axial force caused a fourth metacarpal fracture instead of disruption of the middle–ring metacarpal base joint and a hamate fracture instead of disruption of the capitohamate joint. The force then proceeded to cause the scaphoid to dislocate with the hand in hyperextension and ulnar deviation.

The most common complication after a scaphoid dislocation is scapholunate dissociation, which emphasizes the importance of scapholunate ligament repair or reconstruction in these cases.^7^^,^^16^ In our patient, the scapholunate ligament was not only repaired, but also augmented with FiberTape, which functioned as a short transverse internal brace. The addition of FiberTape has been shown to increase the maximum load to failure compared to solely scapholunate ligament repair.^17^ Moreover, the FiberTape augmentation in our patient was then brought back over to the distal scaphoid pole to form an additional long-oblique internal brace. This combination has been biomechanically advantageous in preventing widening of the scapholunate interval compared with no augmentation or either augmentation method in isolation.^18^ Furthermore, the long-oblique internal brace corrects for dorsal intercalated segment instability and scaphoid rotary subluxation.^18^ Following the scapholunate repair and augmentation in our patient, the secondary stabilizers of the scapholunate joint, including the dorsal intercarpal ligament and dorsal radiocarpal ligament, were also repaired to offer further stability.^19^ At the conclusion of the surgery, there was no persistent dorsal intercalated segment instability deformity or carpal malalignment, confirming the integrity of our procedure.
